# Preparation of *p*-Phenylenediamine Modified Graphene Foam/Polyaniline@Epoxy Composite with Superior Thermal and EMI Shielding Performance

**DOI:** 10.3390/polym13142324

**Published:** 2021-07-15

**Authors:** Liusi Wang, Haoliang Li, Shuxing Xiao, Mohan Zhu, Junhe Yang

**Affiliations:** 1 School of Materials Science and Engineering, University of Shanghai for Science and Technology, No. 516 Jungong Road, Shanghai 200093, China; xuanyunyyds@163.com (L.W.); chhx0920@163.com (S.X.); zhumohan11@163.com (M.Z.); 2 School of Medical Instrument and Food Engineering, University of Shanghai for Science and Technology, No. 516 Jungong Road, Shanghai 200093, China

**Keywords:** graphene foam, in-situ polymerization, conductive network, thermal management, EMI shielding

## Abstract

With the development of integrated devices, the local hot spot has become a critical problem to guarantee the working efficiency and the stability. In this work, we proposed an innovative approach to deliver graphene foam/polyaniline@epoxy composites (GF/PANI@EP) with improvement in the thermal and mechanical property performance. The graphene foam was firstly modified by the grafting strategy of p-phenylenediamine to anchor reactive sites for further in-situ polymerization of PANI resulting in a conductive network. The thermal conductivity (κ) and electromagnetic interference shielding (EMI) performance of the optimized GF/PANI_4:1_@EP is significantly enhanced by 238% and 1184%, respectively, compared to that of pristine EP with superior reduced modulus and hardness. Such a method to deliver GF composites can not only solve the agglomeration problem in traditional high content filler casting process, but also provides an effective way to build up conductive network with low density for thermal management of electronic devices.

## 1. Introduction

With the integration and miniaturization of electronic equipment, heat dissipation in time has become a huge challenge to guarantee the performance and life of the electronic equipment [1,2]. However, the traditional thermal interface materials (TIM), with extremely high loading (up to ~70%) of thermal conductive fillers such as silicone grease or aluminum oxide, can hardly meet the demand, due to the low thermal conductivity (κ, only 1–5 W m^−1^ K^−1^), strong fluidity (pump out leakage from the devices) and the poor dispersion of particle fillers [3,4,5].

Graphene is regarded as one of the promising substitutes for efficient heat dissipation, due to its inherent high κ (~5300 W m^−1^ K^−1^) and large specific surface area of graphene [6,7,8,9]. So far, the research interests of TIMs mainly focused on the influence of preparation methods to the thermal properties of composite materials. Khan M et al. synthesized a multilayer graphene composite with more than 10 vol% loading with an enhancement by 10 times in κ of the pristine matrix [10]. The graphene nanoplatelets (GnP) and cellulose nanocrystals (CNC) hybrid film at high graphene content of 85 wt% was demonstrated with an in-plane and out-of-plane κ of 41 W m^−1^ K^−1^ and 1.2 W m^−1^ K^−1^, respectively [11]. The graphene sheets exfoliated by ball milling was also applied to deliver graphene-epoxy composites, but the κ was only 0.415 W m^−1^ K^−1^, with 2 wt% addition of graphene due to the π–π interaction of graphene sheets [12]. Muhammad RZ et al. reported a solution blending of graphene to increase the κ from 0.21 W m^−1^ K^−1^ to 0.47 W m^−1^ K^−1^ [13]. In fact, in the case of a lower content of filler, it was difficult to form conductive network for electron or phonon transportation for improving the thermal and electrical properties. However, with the increasing amount of filler, due to the poor dispersion of graphene or other fillers, the severe agglomeration led to inferior mechanical properties of the composites [14,15].

To figure out the trade-off problem between the content and the homogeneity of graphene filler, it is an effective method to build up a continuous three-dimensional (3D) filler framework to obtain considerable κ due to its interconnection served as conductive paths for phonon transportation [16,17]. Wei et al. manufactured a reduced GO (rGO) network through freeze-drying method, and the κ of the rGO/PI composite showed an enhancement more than 15 times [18]. The boron nitride is also applied to build up 3D conductive network with PDMS for TIMs with an ultra-high κ of 7.46 W m^−1^ K^−1^ [19]. Although the 3D structure is benefit to the thermal and electrical properties of composite materials, the porosity of these graphene foam will have a negative influence to the κ of the composite due to the severe phonon-interface scattering. Thus, several researches introduced the covalent functionalization of graphene to further improvement. The graphene/Al_2_O_3_ thermal grease was fabricated with only 1 wt% of graphene and exhibited an increasing κ from 2.70 W m^−1^ K^−1^ to 3.45 W m^−1^ K^−1^ [20]. Renteria et al. attached Fe_3_O_4_ nanoparticles onto graphene fillers and induced vertically arrange the hybrid fillers by magnetic field to improve κ of the material [21]. The functionalization of graphene is another effective strategy to provide reactive sites to strengthen the interface interaction between the filler and the matrix, serving as conductive paths for electrons and phonons for heat dissipation. Amine groups have high reactivity and are rich in chemical components, making them easily to react with many other chemical substances. Wang et al. reported the modification of GO by octadecylamine (ODA) to deliver modified graphene oxide/styrene butadiene rubber (GO-ODA/SBR) composites with higher tensile strength and elongation at break due to the covalent crosslink of GO sheets [22]. The p-phenylenediamine (PPD) was also applied to modify the GO filler, with an enhancement in κ to 1.42 W m^−1^ K^−1^ with only 1.5 wt% of filler loading [23].

In this study, an innovative method is approached to prepare graphene foam/polyaniline@epoxy (GF/PANI@EP) with conductive network, as well as a lower content of filler. The graphene foam is firstly modified by p-phenylenediamine (PPD) to anchor the reactive sites for further in-situ polymerization of PANI to build up a connective network. The κ shows a significant increase by 238%, with only 1 wt% of graphene filler loading. The electromagnetic interference (EMI) shielding performance with 38.32 dB, 11 times larger than that of the pristine matrix. Moreover, the mechanical property is also enhanced to 4.039 GPa due to covalent interaction between PANI and GF achieved by in-situ polymerization. Such an innovative method provides an effective way to prepare TIMs for the thermal management of the next generation devices.

## 2. Materials and Methods

### 2.1. Materials

Graphite power (200 mesh), *p*-phenylenediamine, and hydrazine hydrate were purchased from Aladdin (Shanghai, China). Hydrochloric acid, aniline, ammonium persulfate, potassium permanganate, sulfuric acid (98%), nitric acid (65%), hydrogen peroxide (30%), ferric chloride hexahydrate, and ethanol were all purchased from National Pharmaceutical Group Chemical (Shanghai, China) Co., Ltd. Epoxy resin and curing agent, provided by South Asia Electronic Materials (Kunshan, China).

### 2.2. The Modification of PPD and the Preparation of GF/PANI Filler

Graphene oxide (GO) was prepared by modified Hummers method without sonication treatment. The modification of PPD was achieved by a feasible solution blending method. Concretely, 26 mg PPD is dissolved in 4 mL deionized water and heat it at 80 °C until it is completely dissolved. Then, the 26 mL GO aqueous solution (5 mg mL^−1^) and PPD aqueous solution were mixed under stirring for 30 min. Then the solution was transferred into a hydrothermal reactor, heated to 150 °C for 12 h to obtain GF/PPD. The as-obtained was named as GF/PPD_5:1_ (mass ratio of GO to PPD was 5:1). The GF/PPD_3:1_ and GF/PPD_4:1_ were prepared in the same manner. Then, GF/PPD was immersed in ethanol solution, 390 μL hydrazine hydrate were added ethanol solution, and GF/PPD was further reduced by microwave (100 W, 120 °C, 30 min). In a typical process, the 60 μL aniline (AN) monomer was dispersed in a solution of HCl (24 mL) and sonicated for 15 min until the monomer was completely dispersed. GF/PPD_5:1_ was immersed in the above solution in an ice bath (<4 °C) and stirred for 30 min. Ammonium persulfate solution (0.063 g) was slowly dropped, the mixture was continuously stirred for 4 h (ice bath), the reaction solution turned dark green, and the product GF/PANI_5:1_ was obtained. The as-obtained was named as GF/PANI_5:1_ (mass ratio of GO to AN was 5:1). The GF/PANI_3:1_ and GF/PANI_4:1_ were prepared in the same manner. Finally, the GF/PANI_5:1_ was freeze-dried for 48 h.

### 2.3. Preparation of GF/PANI@EP Composite

**Figure 1:**
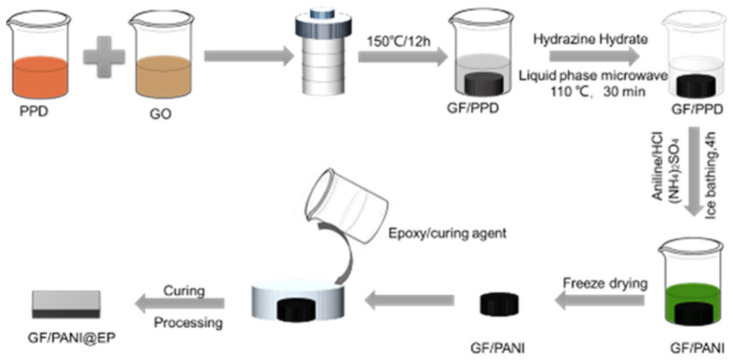
Schematic diagram of the fabrication for PPD modified GO/polyaniline @ EP thermal interface materials.

The GF/PANI@EP composite was prepared as follows. Firstly, the EP and curing agent (4:1, *w*:*w*) were stirred at room temperature for 30 min. Then the filler is immersed into the matrix and followed by curing in an oven at 80 °C for 40 min, as shown in Figure 1. The as-obtained composite material is denominated as GF/PANIx:1@EP, according to the weight ratio between GO and PANI.

### 2.4. Characterization

Fourier transform infrared spectroscopy (FTIR, P.E. Spectrum100, Wellesley, MA, USA) was collected on a spectrum from 800 cm^−1^ to 4000 cm^−1^ at a resolution of 2 cm^−1^ with an accumulation of 8 times. Raman spectra (Horiba, LabRAM HR Evolution, Kyoto, Japan) were collected from 600 cm^−1^ to 2000 cm^−1^ with a laser length at 532 nm. X-ray photoelectron spectra (XPS, Physical Electronics PHI 5000C ESCA, Saint Vincent, Italy) were performed to acquire the chemical bonding and the valence states of GF, GF/PPD, and GF/PANI with a monochromatized Al Kα X-ray source (1486.71 eV). X-ray diffraction (XRD, Bruker D8 ADVANCE, Munich Germany) patterns were conducted using nickel-filtered Cu Kα (k ¼ 0.154 nm) radiation with a generator voltage of 40 kV and a current of 40 mA. The scanning speed was 5° min^−1^ and the step size was 0.02° with the range from 5° to 60°. The surface and cross-section morphology of samples were observed by scanning electron microscopy (SEM, FEI Quanta FEG, Hillsboro, OR, USA) with an accelerating voltage of 20 kV. The elemental distribution mapping of GF/PANI_4:1_ was measured by energy dispersive spectrometer (EDS, Oxford Instruments, Oxford, UK). The electrical conductivity of GF@EP, GF/PPD@EP, GF/PANI@EP was measured by in-line four-point probe method (RTS-9, Scientific Equipment and Services, Cambridge, MA, USA) at room temperature, and the thickness of each sample was 4 mm. The current range during the test is 10 mA. The mechanical properties of the samples can be measured by the NANO instrument (Agilent, Palo Alto, CA, USA), a G200 nano indenter with the Berkovich indenter. The load-nload curves were obtained by accurately recording the change of the loading depth with the load. The hardness and elastic modulus were calculated by the Oliver-Pharr model. The thermal diffusivity (α) of composite materials was measured by laser flash analyzer (LFA 467/Ins Nanoflash, Netzsch, Selb/Bavaria, Germany) at room temperature. The κ was calculated by the Equation (1):

κ = Cp × ρ × α(1)

where Cp and ρ represents the specific heat, density and thermal diffusivity of the sample, respectively. All samples were cut into a diameter of 12.7 mm with a thickness of ~4 mm.

The electromagnetic interference (EMI) shielding properties of the composite films were performed in the frequency range of 8.2 GHz to 12.4 GHz (X band) using the waveguide method via the vector network analyzer (Agilent N5222B, Palo Alto, CA, USA). All samples were cut into rectangle plates with a size of 22.8 mm × 10.1 mm × 4 mm to fit the waveguide sample holder. The scattering parameters (S11 and S21) of the GF/PANI@EP composite materials were collected to calculate the EMI shielding effectiveness.

## 3. Results and Discussion

### 3.1. Structure and Characterization

**Figure 2:**
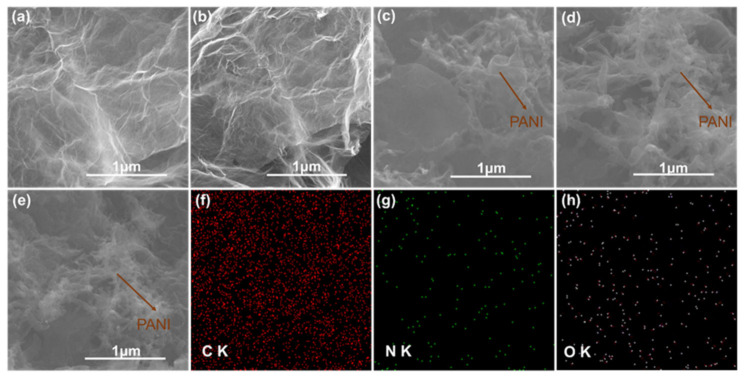
SEM images of the (**a**) GF, (**b**) GF/PPD, (**c**) GF/PANI_5:1_, (**d**) GF/PANI_3:1_, (**e**) GF/PANI_4:1_ and (**f**) C, (**g**) N, and (**h**) O element mapping of GF/PANI_4:1_.

The morphology of GF, GF/PPD, and GF/PANI fillers is shown in Figure 2. Compared with the surface of GF, more wrinkles can be observed in the GF/PPD suggesting that PPD was successfully grafted onto GO. After the in-situ polymerization, as displayed in Figure 2c–e, the fibrous PANI is attached onto the surface of GF/PANI filler. Moreover, both the SEM image and the EDX mapping of GF/PANI_4:1_ in Figure 2e–h exhibits a uniform distribution of PANI covered on the graphene sheets forming a continuous network. However, the entanglement of fibers gets intensified with an increasing width in GF/PANI_3:1_, indicating the overdose of AN will lead to serious agglomeration [24,25]. Additionally, according to the electrical conductivity (σ) of each filler in Figure S1a, the GF/PANI_4:1_ also exhibits the highest σ up to 32.58 S m^−1^, suggesting the presence of fibrous conductive PANI has successfully connected the graphene sheets to provide effective pathways for electron transportation.

The morphology difference between the graphene/PANI filler and its epoxy composites is shown in Figure S2. Both the GF/PPD and the GF/PANI filler exhibit a highly porous structure, but after the infiltration of matrix, the porosity of the GF/PANI@EP on the surface is obviously lower than that of the GF@EP and GF/PPD@EP, respectively, suggesting the in-situ polymerization of PANI benefits the interaction between the filler and epoxy matrix. As listed in Figure S1b, the σ of GF@EP is significantly improved to 17.67 S m^−1^, over 5 times higher compared to that of the pristine EP matrix (only 3.00 S m^−1^). While the highest σ of GF/PANI_4:1_@EP increases to 28.52 S m^−1^, suggesting the presence of PANI has connected the graphene sheets and served as conductive pathway.

**Figure 3:**
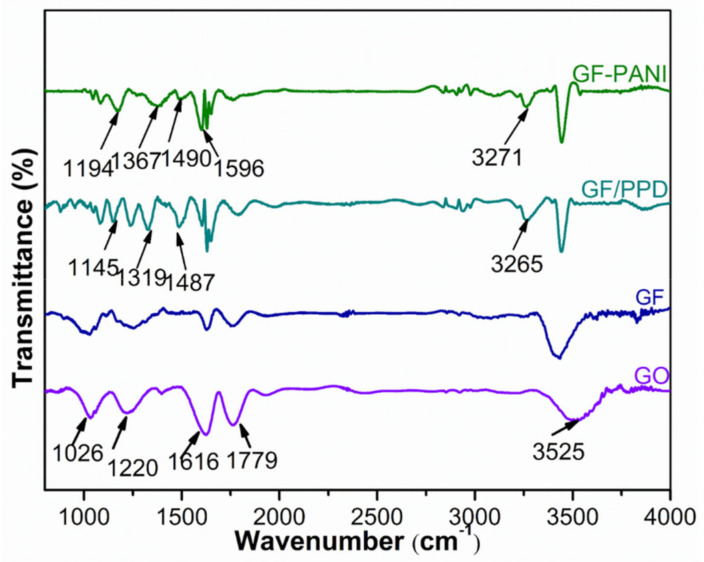
FTIR spectra of the GO, GF, GF/PPD, and GF/PANI.

Figure 3 represents the FTIR spectrum of GO, GF, GF/GF/PPD, and GF/PANI filler. The spectra for GO show a typical broad absorption band at 3525 cm^−1^, which is assigned to the stretching vibration of the hydroxyl groups [26]. The peaks at 1779 cm^−1^, 1616 cm^−1^, 1220 cm^−1^, and 1026 cm^−1^ corresponded to the C=O stretching vibration in carboxylic groups, bending vibration of hydroxyl and the stretching vibration of epoxide and alkoxy in GO, respectively [27,28]. After the hydrothermal process to obtain GF, the peak intensity at 1779 cm^−1^ and 1616 cm^−1^ get decreased, indicating the partially reduction in GO. Moreover, the GF/PPD exhibits three additional peaks at 3265 cm^−1^, 1487 cm^−1^, and 1145 cm^−1^, corresponding to the vibration of N–H and C–N groups, respectively, suggesting that the PPD has successfully grafted onto the GO sheets [29,30]. The spectra of the GF/PANI, located at 1596 cm^−1^, 1490 cm^−1^, 1367 cm^−1^, and 1194 cm^−1^ corresponding to the benzenoid or quinonoid ring vibrations and C–N or C=N vibration, respectively [31], indicating that the PPD anchored onto the GO sheets is served as reactive sites for the in-situ polymerization of PANI resulting in a conductive network.

**Figure 4:**
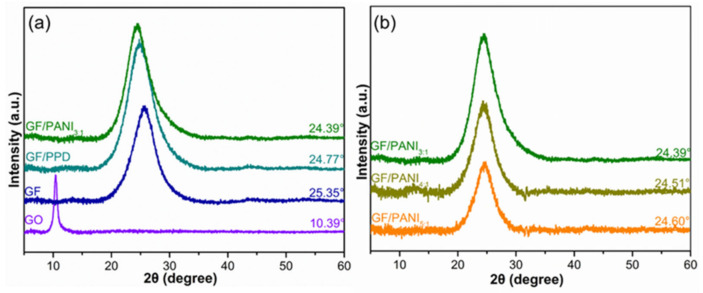
The XRD patterns of (**a**) GO, GF, GF/PPD, GF/PANI_3:1_ and (**b**) GF/PANI_5:1_, GF/PANI_4:1_, GF/PANI_3:1_ filler with different amount of PANI.

Figure 4a exhibits the XRD patterns of the GO, GF, GF/PPD, and GF/PANI. The diffraction peak of GF dramatically shifts to 25.35° (vs. 10.39°) corresponding to the interlayer spacing of 0.3509 nm, due to the removal of oxygen-containing functional groups and the restoration of π–π conjugated carbon network during the hydrothermal process [32]. While the diffraction peak of GF/PPD has swiftly moved to 24.77° corresponding to the d_002_ of 0.3590 nm, suggesting that the PPD has successfully grafted onto the GO by chemical cross-linking [33]. The GF/PANI_3:1_ shows an obvious diffraction peak at 24.39° corresponding to the d_002_ of 0.3645 nm, revealing the incorporation of PANI has in-situ synthesized and expanded the interlayer space of GF. Besides, as shown in Figure 4b and Table S1, with the increasing amount of PANI, the interlayer distance of GF/PANI gradually increases. The d_002_ exhibits a gradually upward trend with the increasing amount of PANI to 0.3615 nm, 0.3628 nm, and 0.3645 nm corresponding to GF/PANI_5:1_, GF/PANI_4:1_, and GF/PANI_3:1_, respectively, suggesting that the PANI has in-situ polymerized and welded graphene sheets up forming an interconnected network.

**Figure 5:**
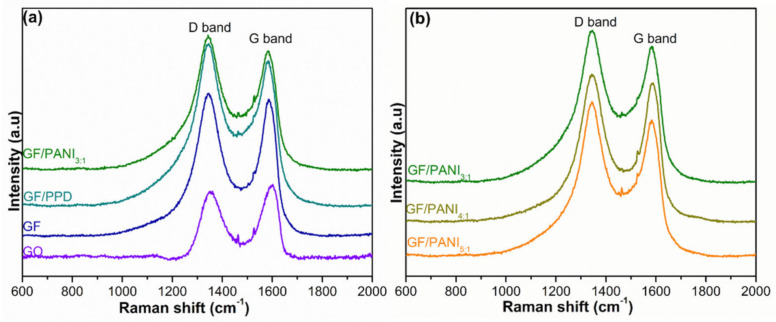
The Raman spectroscopy of (**a**) GO, GF, GF/PPD, GF/PANI_3:1_ and (**b**) GF/PANI_5:1_, GF/PANI_4:1_, GF/PANI_3:1_ filler with different amount of PANI.

The Raman spectrum of GO, GF, GF/PPD, and GF/PANI is displayed in Figure 5. The D band (at ~1353 cm^−1^) is attributed to A_1g_ phonons of disordered structure, while the G band (at ~1587 cm^−1^) is associated with the E_2g_ vibration of sp^2^ hybrid C–C bonding [28]. The peak position exhibits a swiftly blue shift, and the intensity ratio (I_D_/I_G_) of GF shows an increase to 1.026 (vs. 0.867) suggesting the reduction process alters the structural integrity of the honeycomb carbon network. After the modification by PPD, the I_D_/I_G_ gets a further increase to 1.115, suggesting that the covalent welding of the –NH_2_ moiety on the GF [33,34]. The I_D_/I_G_ of GF/PANI_3:1_ shows a further increase to 1.158. Moreover, as displayed in Figure 5b, the I_D_/I_G_ exhibits a gradually upward trend with the increasing amount of PANI to 1.121, 1.138, and 1.158 corresponding to GF/PANI_5:1_, GF/PANI_4:1_, and GF/PANI_3:1_, respectively, indicating that the PANI has successfully in-situ polymerized and formed interconnection network in GF, which provides an effective pathway for phonon transportation during the heat dissipation process. The result is consistent with the XRD result.

**Figure 6:**
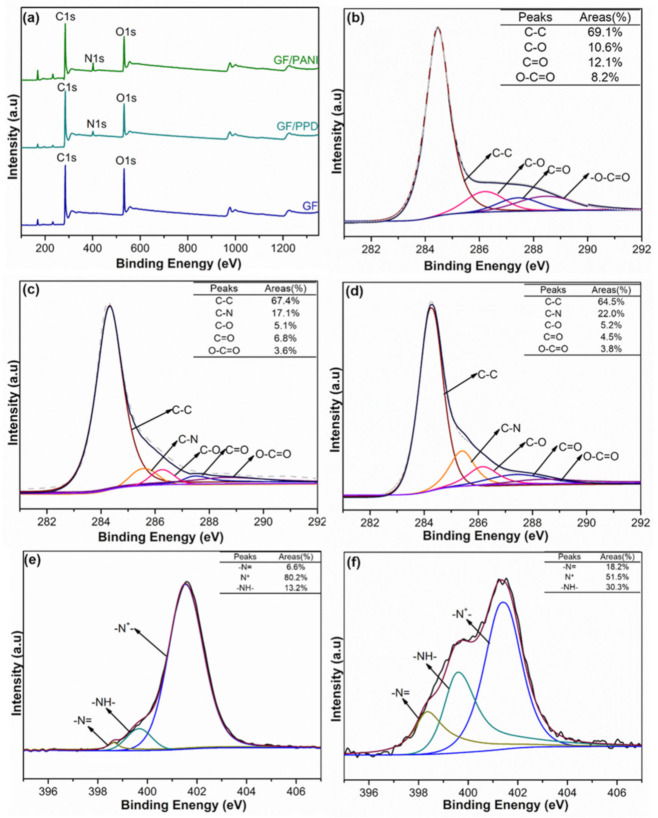
(**a**) The full XPS spectra of GF, GF/PPD, and GF/PANI membranes. C 1s spectrum of (**b**) GF, (**c**) GF/PPD, and (**d**) and GF/PANI; N 1s spectrum of (**e**) GF/PPD and (**f**) GF/PANI.

Figure 6a represents the XPS spectra to investigate the valance state of C and N element of GF, GF/PPD, and GF/PANI filler. The high resolution C1s spectrum of GF, as shown in Figure 6b, can be assigned to four prominent peaks at 284.3 eV, 286.2 eV, 287.5 eV, and 288.6 eV, corresponding to C=C, C–O, C=O, and O–C=O bonding, respectively [35,36]. In Figure 6c, an additional peak attributed to C–N group at 285.5 eV is observed in GF/PPD suggesting the successful grafting modification of PPD on to the graphene sheets. Moreover, the content of each group is dramatically decreased to 5.1% (vs. 10.6%), 6.8% (vs. 12.1%), and 3.6 (vs. 8.2%), respectively, suggesting these oxygen containing groups in GF can serve as potential reaction site for the modification process, which is consistent with the FT-IR spectrum. After the in-situ polymerization of PANI in Figure 6d, the content of C–N group in GF/PANI filler increases to 22.0% (vs. 17.1% of GF/PPD), while the content of C–O and O–C=O groups is almost same to the GF/PPD, indicating the priority of in-situ synthesis of PANI at the grafted PPD as reactive sites rather than the oxygen groups on graphene sheets. Furthermore, in Figure 6e,f, the N 1s spectra can be divided into 3 components, corresponding to quinoid imine (–N= generated between C=O and –NH2), secondary amine (–NH– in –NH–CO– groups) and protonated nitrogen (–N+–) groups, respectively. Hence, the increasing atomic ratio of –N= and –NH– and the decreasing ratio of –N+– can be ascribed to the successfully in-situ polymerization of PANI at the grafting PPD as reactive sites [37,38].

### 3.2. Thermal Conductivity and Mechanical Properties

**Figure 7:**
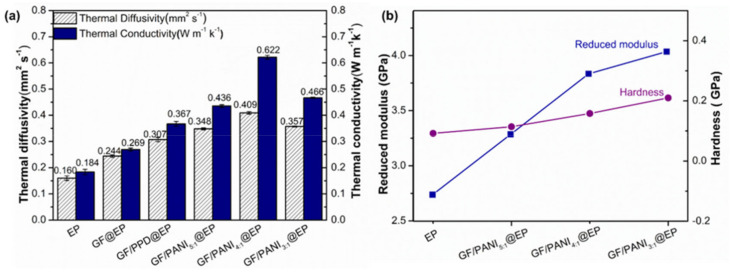
(**a**) The thermal conductivity (κ) and (**b**) The hardness and reduced modulus of the EP, GF/PANI_5:1_@EP, GF/PANI_4:1_@EP, GF/PANI_3:1_@EP nanocomposites.

The κ of GF@EP, GF/PPD@EP and GF/PANI@EP with different PANI loading is shown in Figure 7a. In comparison with the neat epoxy resin, the κ of GF@EP is enhanced to 0.269 W m^−1^ K^−1^ (vs. 0.184 W m^−1^ K^−1^), suggesting 3D graphene foam network affords effective pathway for heat transfer in polymer matrix [39]. After the modification of PPD, the κ of GF/PPD@EP shows only a slight increase to 0.367 W m^−1^ K^−1^. Notably, with the further in-situ polymerization of PANI, the κ of GF/PANI_5:1_@EP and GF/PANI_4:1_@EP is further improved to 0.436 W m^−1^ K^−1^ and 0.622 W m^−1^ K^−1^, respectively. Because the graphene sheets have been connected by the presence of PANI during the in-situ polymerization process forming a continuous network for phonon transportation pathway to improve the thermal properties [23]. However, with the overload of PANI to 33% (GF/PANI_3:1_@EP), the κ displays a dramatic decrease to 0.466 W m^−1^ K^−1^ due to the entanglement and the agglomeration of excessive PANI in the composites. Although the κ is still lower than that of the reported TIMs, such as PDMS composites [19] or thermal grease [20], the 3D conductive network achieved by such a modification and in-situ polymerization of PANI is benefit to the heat dissipation performance. The reduced modulus (Er) and hardness of pure epoxy and the GF/PANI@EP composites with different loading of PANI is shown in Figure 7b. The Er and the hardness of EP is only 2.739 GPa and 0.092 GPa, respectively. With the in-situ polymerization of PANI, the Er and the hardness of GF/PANI_5:1_@EP and GF/PANI_4:1_@EP shows a liner increase to 3.285 GPa, 0.114 GPa and 3.835 GPa, 0.158 GPa, respectively. The result indicates that the network formed by the PANI connection will strengthen the interaction between the GO sheet and the polymer matrix, leading to the increasing Er and hardness [40].

### 3.3. Electromagnetic Shielding Performance

**Figure 8:**
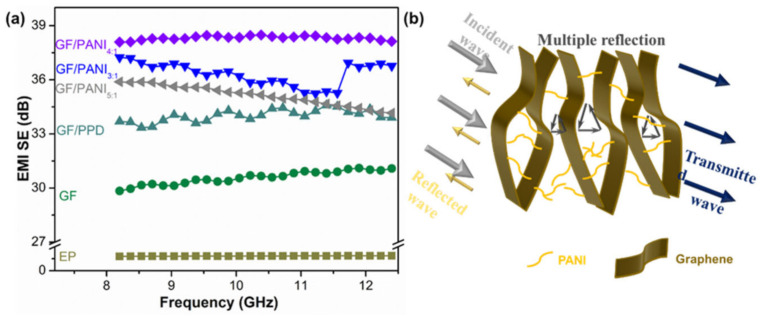
(**a**) EMI SE values of the f the EP, GF@EP, GF/PPD@EP GF/PANI_5:1_@EP, GF/PANI_4:1_@EP, GF/PANI_3:1_@EP nanocomposites and (**b**) schematic illustration of EMI shielding mechanism.

Figure 8 depicts the EMI SE values of EP, GF@EP, GF/PPD@EP, and GF/PANI@EP composites in the X-band. The pristine EP exhibit a relatively poor EMI shielding performance with only 3.06 dB. The EMI SE value of GF@EP is increased to 30.58 dB. Because the conductive network of GF could attenuate electromagnetic radiation easily due to reflection and scattering microwave, and incident electromagnetic micro-waves are transferred to heat by being absorbed. In comparison with the GF@EP, the GF/PPD@EP shows a slight enhancement to 34.01 dB, due to the covalent functionalization partially reduction in GO with an increasing electrical conductivity. However, after the in-situ polymerization of PANI, the GF/PANI@EP composites display a further improvement in EMI shielding performance to 35.13 dB and 38.32 dB corresponding to GF/PANI_3:1_@EP and GF/PANI_4:1_@EP, respectively, suggesting the PANI has connected the graphene sheets in GF and forming a continuous network for electron transportation. However, with the overdose of PANI to a ratio of 3:1, the EMI shielding performance of GF/PANI_3:1_@EP has an obvious decline to 36.33 dB, due to the agglomeration of PANI. The formation of conductive networks within the EP matrix play an important role in the EMI shielding performance of the composite. Figure 8b shows the EMI shielding mechanism of GF/PANI@EP. A portion of electromagnetic wave is reflected at the interface of the GF/PANI@EP, because conductive networks with a large amount of charge carriers are beneficial to EMI shielding effectiveness by refection. The remaining portion penetrating the surface is multiply reflected or absorbed by the 3D conductive network [41]. Only a small amount of electromagnetic waves pass through the composite. The formation of an excellent conductive network between GF and PANI in EP matrix ensures that the electromagnetic wave is greatly reduced, leading to an outstanding EMI shielding performance [42].

### 3.4. Mechanism Diagram

**Figure 9:**
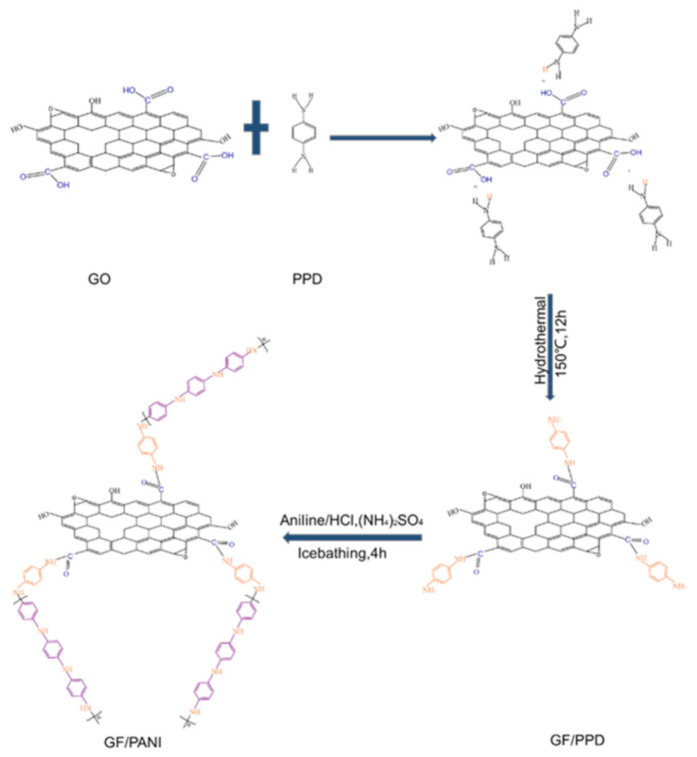
Mechanism diagram of *p*-phenylenediamine modified GO/polyaniline.

The mechanism diagram of the modification of PPD and the further in-situ molecular welding of GF/PANI composite is illustrated in Figure 9. The nature of the grafting modification of GO is contributed to the nucleophilic substitution reaction between the amine group in PPD and the carboxyl group on the surface of GO forming covalent –CONH bonding. The presence of PPD served as reactive sites for the further in-situ polymerization of PANI resulting in a continuous network, provides effective pathway for phonon or electron transportation [31,43]. However, when the PANI is excessive, the entanglement of PANI fiber will introduce stronger phonon scattering, which hinder the heat dissipation performance.

## 4. Conclusions

In conclusion, we have reported a novel method to prepare GF/PANI@EP composite with a low filler content (1 wt%), but thermal conductive networks with enhanced properties. The graphene foam modified by PPD provides sufficient reactive sites for further in-situ polymerization of PANI forming continuous network for phonon transportation. When the mass ratio of GO to aniline is 4:1, the GF/PANI_4:1_@EP exhibits a superior κ of 0.622 W m^−1^ K^−1^, 3 times larger than that of the pristine matrix. At the same time, the EMI shielding performance of GF/PANI_4:1_@EP reached to 38.32 dB and the reduced modulus and hardness improves by 2 times to 4.036 GPa and 0.210 GPa, respectively. The modified method in this work to fabricate graphene based fillers with conductive network shows great potential for TIMs and EMI shielding performance.

## Data Availability

If you need to have access to some of the raw data, please contact the corresponding author.

## Supplementary Materials

The following are available online at https://www.mdpi.com/article/10.3390/polym13142324/s1, Figure S1: Electrical conductivities of the(a) GF, GF/PPD, GF/PANI_5:1_, GF/PANI_4:1_ GF/PANI_3:1_. and (b) GF@EP, GF/PPD@EP GF/PANI_5:1_@EP, GF/PANI_4:1_@EP, GF/PANI_3:1_@EP nanocomposites. Table S1: 2θ and d002 of GO, GF, GF/PPD, GF/PANI_5:1_, GF/PANI_4:1_ and GF/PANI3:1, Table S2: I_D_/I_G_ of GO, GF, GF/PPD, GF/PANI_5:1_, GF/PANI_4:1_ and GF/PANI_3:1_. Figure S2: SEM images of the(a) GF, (b) GF/PPD, (c) GF/PANI_4:1_, (d) GF@EP, (e) GF/PPD@EP and (f) GF/PANI@EP
