# Supplementary material for: Preparation of p-Phenylenediamine Modified Graphene Foam/Polyaniline@Epoxy Composite with Superior Thermal and EMI Shielding Performance
Source: Polymers (Basel). 2021 Jul 15;13(14):2324. doi: 10.3390/polym13142324 (PMC8309473; doi:10.3390/polym13142324)
Supplement: Supplementary file 1 [file polymers-13-02324-s001.zip › polymers-1185572-supplementary.pdf]

Supporting information

Preparation of p-phenylenediamine modified graphene foam/polyaniline@epoxy composite for thermal management and EMI shielding

Liusi Wang [1], Haoliang Li [1,2,*], Shuxing xiao [1], Mohan Zhu [1], and Junhe Yang [1,*]

[1]School of Materials Science and Engineering, University of Shanghai for Science and Technology, No.516 Jungong Road, Shanghai 200093, China;
[2]School of Medical Instrument and Food Engineering, University of Shanghai for Science and Technology, No.516 Jungong Road, Shanghai 200093, China;

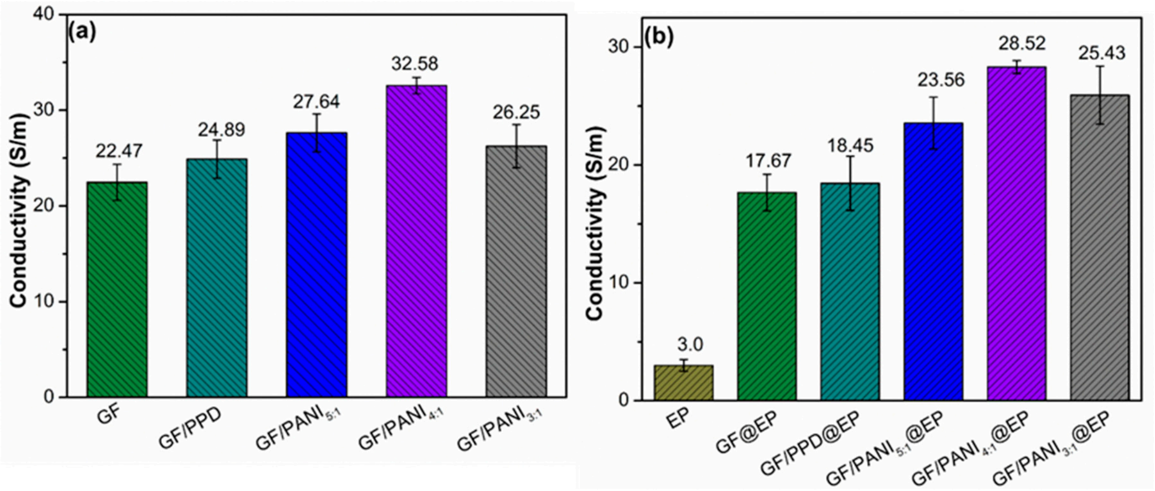

Figure S1. Electrical conductivities of the(a) GF, GF/PPD, GF/PANI$_{5:1}$, GF/PANI$_{4:1}$ GF/PANI$_{3:1}$. and (b) GF@EP, GF/PPD@EP GF/PANI$_{5:1}$@EP, GF/PANI$_{4:1}$@EP, GF/PANI$_{3:1}$@EP nanocomposites.

Table S1 2θ and d_{002} of GO, GF, GF/PPD, GF/$PANI_{5:1}$, GF/$PANI_{4:1}$ and GF/$PANI_{3:1}$

sample	2θ°	d_{002} (nm)
GO	10.39°	0.8504
GF	25.35°	0.3509
GF/PPD	24.77°	0.3590
GF/$PANI_{5:1}$	24.60°	0.3615
GF/$PANI_{4:1}$	24.51°	0.3628
GF/$PANI_{3:1}$	24.39°	0.3645

Table S2 I_D/I_G of GO, GF, GF/PPD, GF/$PANI_{5:1}$, GF/$PANI_{4:1}$ and GF/$PANI_{3:1}$

sample	I_D/I_G
GO	0.867
GF	1.026
GF/PPD	1.115
GF/$PANI_{5:1}$	1.121
GF/$PANI_{4:1}$	1.138
GF/$PANI_{3:1}$	1.158

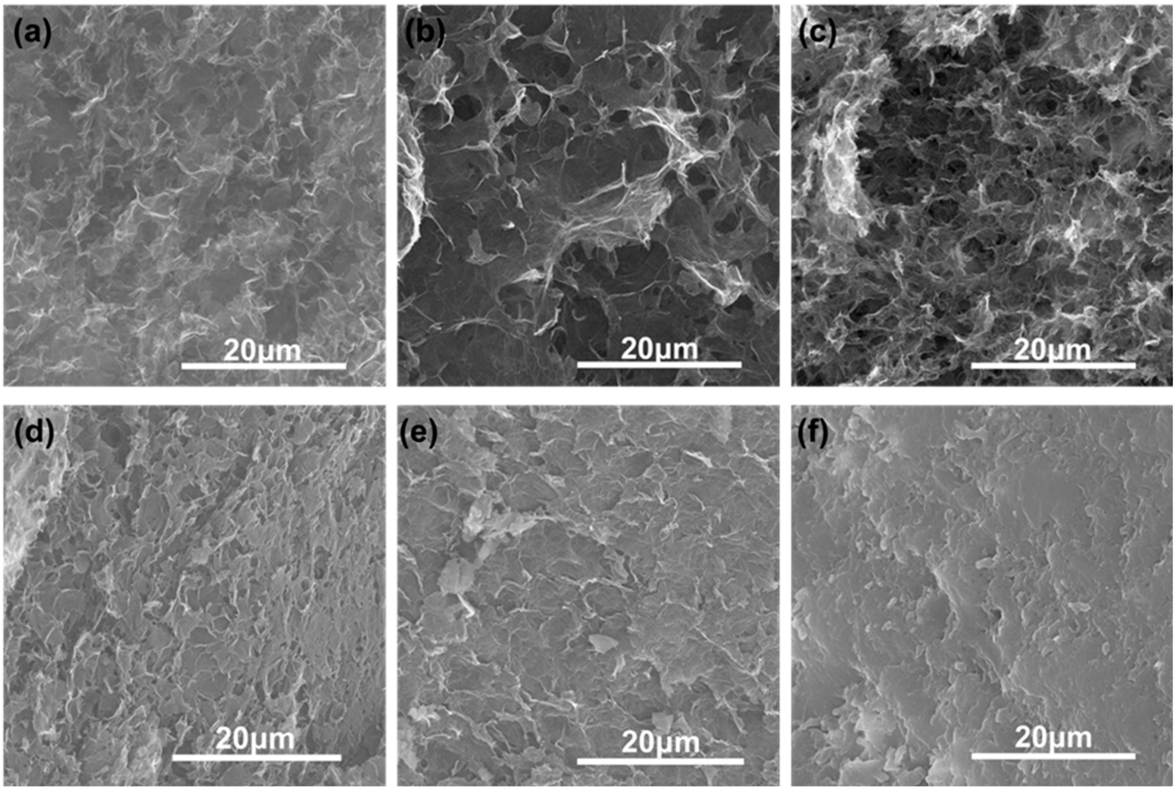

Figure S2. SEM images of the(a) GF, (b) GF/PPD, (c) GF/$PANI_{4:1}$, (d) GF@EP, (e) GF/PPD@EP and (f) GF/PANI@EP

Table S3 Comparison of thermal conductivity of this work with the previously reported GNP-based polymer composites.

sample	Full loading	TC (W m^{-1} K^{-1})	TC enhancement (%)	Reference
Graphene/MLG@EP	2 wt%	14	141%	[1]
GNPRE	2 wt%	0.415	88%	[2]
GnP/CNC	85 wt%	41	310%	[3]
GNPs/Al_2O_3@ silicone base	1 wt%	3.450	28%	[4]
GNP/EP	3 wt%	0.470	126%	[5]
rGO/PI	8 wt%	2.780	1500%	[6]
BNNS/PDMS	15.8 vol%	7.460	3900%	[7]
GF/PANI@EP	1 wt%	0.622	238%	This work

1. Shahil, K. M. F.; Balandin, A. A. Graphene-multilayer graphene nanocomposites as highly efficient thermal interface materials. *Nano Lett.* **2012**, 12, 861-867.
2. Ahmadi-Moghadam, B.; Taheri, F. Effect of processing parameters on the structure and multi-functional performance of epoxy/GNP-nanocomposites. *J. Mater. Sci.* **2014**, 49, 6180-6190.
3. Wang, F.; Drzal, L. T.; Qin, Y.; Huang, Z. Multifunctional graphene nanoplatelets/cellulose nanocrystals composite paper. *Compos. Part B-Eng.* **2015**, 79, 521-529.
4. Yu, W.; Xie, H.; Yin, L.; Zhao, J.; Xia, L.; Chen, L. Exceptionally high thermal conductivity of thermal grease: Synergistic effects of graphene and alumina. *Int. J. Therm. Sci.* **2015**, 91, 76-82.
5. Zakaria, M. R.; Abdul Kudus, M. H.; Md. Akil, H.; Mohd Thirmizir, M. Z. Comparative study of graphene nanoparticle and multiwall carbon nanotube filled epoxy nanocomposites based on mechanical, thermal and dielectric properties. *Compos. Part B-Eng.* **2017**, 119, 57-66.
6. Wei, S.; Yu, Q.; Fan, Z.; Liu, S.; Chi, Z.; Chen, X.; Zhang, Y.; Xu, J. Fabricating high thermal conductivity rGO/polyimide nanocomposite films via a freeze-drying approach. *RSC. Adv.* **2018**, 8, 22169-22176.
7. Hou, X.; Chen, Y.; Lv, L.; Dai, W.; Zhao, S.; Wang, Z.; Fu, L.; Lin, C. T.; Jiang, N.; Yu, J. High-thermal-transport-channel construction within flexible composites via the welding of boron nitride nanosheets. *ACS Appl. Nano Mater.* **2019**, 2, 360-368.
